# Crystal structure of bis­[bis­(1,4,7-tri­aza­cyclo­nonane-κ^3^ *N*,*N*′,*N*′′)chromium(III)] tris­(tetra­chlorido­zincate) monohydrate from synchrotron X-ray data

**DOI:** 10.1107/S2056989019003086

**Published:** 2019-03-07

**Authors:** Dohyun Moon, Jong-Ha Choi

**Affiliations:** a Pohang Accelerator Laboratory, POSTECH, Pohang 37673, Republic of Korea; b Department of Chemistry, Andong National University, Andong 36729, Republic of Korea

**Keywords:** crystal structure, 1,4,7-tri­aza­cyclo­nona­ne, tetra­chlorido­zincate, chromium(III) complex, hydrogen bonding, synchrotron radiation

## Abstract

The structure of the title compound, [Cr(tacn)_2_]_2_[ZnCl_4_]_3_·H_2_O (tacn is 1,4,7-tri­aza­cyclo­nonane; C_6_H_15_N_3_), has been determined from synchrotron X-ray data. Each Cr^III^ cation is coordinated by the six N atoms from the two tacn ligands, displaying a distorted octa­hedral geometry. Three distorted tetra­hedral [ZnCl_4_]^2−^ anions and one lattice water mol­ecule lie outside this coordination sphere. The Cr—N bond lengths are in the range 2.0621 (11) to 2.0851 (12) Å, while the mean inner N—Cr—N bond angle is 82.51 (5)°. The crystal packing is stabilized by hydrogen-bonding inter­actions with the N—H groups of the tacn ligands and the water O—H groups acting as donors, and the O atoms of the water mol­ecules and Cl atoms of the [ZnCl_4_]^2−^ anions as acceptors. Overall these contacts lead to the formation of a three-dimensional network.

## Abstract 2

Each Cr^III^ cation in the title compound is coordinated by the six N atoms from two 1,4,7-tri­aza­cyclo­nonane (tacn) ligands, displaying a distorted octa­hedral environment. The crystal packing is stabilized by extensive hydrogen-bonding inter­actions involving the N—H groups of the tacn ligands, O—H groups or O atoms of the water mol­ecules and Cl atoms of the [ZnCl_4_]^2−^ anions.

## Chemical context   

The 1,4,7-tri­aza­cyclo­nonane (tacn, C_6_H_15_N_3_) ligand can coordinate facially to many transition metal ions in various oxidation states (Chaudhuri & Wieghardt, 1987 ▸). The macrocycle tacn is tridentate, a pure σ-donor with no π-acceptor capability. In particular, the preparation, spectroscopic properties and ligand field analysis of a [Cr(tacn)_2_]^3+^ complex with a chloride anion have been described (Wieghardt *et al.*, 1983 ▸; Lee & Hoggard, 1991 ▸). Counter-anionic species play very important roles in the coordination chemistry and supra­molecular chemistry of such complexes (Fabbrizzi & Poggi, 2013 ▸; Santos-Figueroa *et al.*, 2013 ▸). The crystal structure of [Cr(tacn)_2_]Br_5_·5H_2_O (Scarborough *et al.*, 2011 ▸) has been reported, but a [Cr(tacn)_2_]^3+^ complex with a [ZnCl_4_]^2−^ counter-anion is not known.
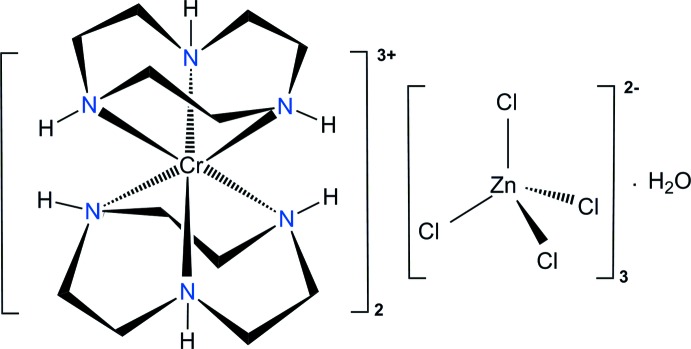


The title compound is another example of a [Cr(tacn)_2_]^3+^ complex but with a different counter-anion. In order to confirm that the crystal is a salt of the [ZnCl_4_]^2−^ anion, we report here the mol­ecular and crystal structure of the new complex [Cr(tacn)_2_]_2_[ZnCl_4_]_3_·H_2_O, (I) determined from synchrotron X-ray data.

## Structural commentary   

**Figure 1:**
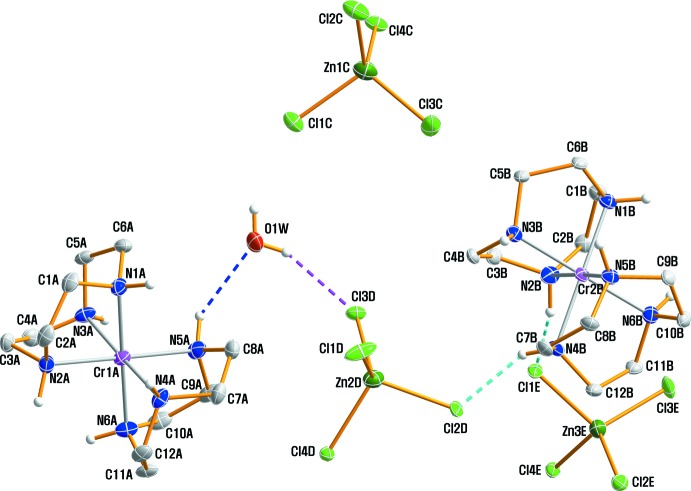
The structures of the mol­ecular components in the asymmetric unit of the title complex (I), drawn with displacement ellipsoids at the 70% probability level. Dashed lines represent hydrogen-bonding inter­actions. The H atoms on the C atoms have been omitted for clarity.

The X-ray structural determination of (I) was carried out at 100 (2) K with synchrotron radiation to confirm its exact geometry and composition. The structure consists of two independent [Cr(tacn)_2_]^3+^ cations, three [ZnCl_4_]^2−^ anions and one lattice water mol­ecule. Fig. 1 ▸ shows an ellipsoid plot of the asymmetric unit of compound (I) with the atomic labelling scheme. The Cr^III^ cation in both [Cr1*A*(tacn)_2_]^3+^ and [Cr2*B*(tacn)_2_]^3+^ is coordinated by the six N atoms from the two tacn ligands, displaying a distorted octa­hedral geometry. The Cr—N(tacn) bond distances for [Cr1*A*(tacn)_2_]^3+^ and [Cr2*B*(tacn)_2_]^3+^ are in the ranges 2.0709 (11) to 2.0828 (11) Å and 2.0621 (11) to 2.0851 (11) Å, respectively, in good agreement with the observed values in [Cr(tacn)_2_]Br_3_·5H_2_O [2.073 (1) Å; Scarborough *et al.*, 2011 ▸] and [Cr(chxn)_3_][ZnCl_4_]Cl·3H_2_O [2.0737 (12)–2.0928 (12) Å; chxn = *trans*-1,2-cyclohexanediamine, C_6_H_14_N_2_; Moon & Choi, 2016 ▸]. However, the bond lengths and bond angles of the two discrete [Cr(tacn)_2_]^3+^ cations are slightly different from each other. In general, three metrics of the bond angles for [*M*(tacn)_2_]^n+^ cations are used. The angles are N—*M*—N_intra_ for the intra­ligand angles, and N—*M*—N_trans_ and N—*M*—N_inter_ for *trans* and *cis* inter­ligand angles, respectively (Lord *et al.*, 2009 ▸). The mean N—*M*—N_intra_, N—*M*—N_trans_ and N—*M*—N_inter_ for [Cr1*A*(tacn)_2_]^3+^ are 82.35 (5), 178.60 (5) and 97.64 (5)° while the three corres­ponding angles for [Cr2*B*(tacn)_2_]^3+^ are 82.66 (5), 177.13 (5) and 97.36 (5)°, respectively. These values for each of the three types of angles may be compared with the literature values for [*M*(tacn)_2_]^n+^ (*M* = Mn^2+^, Fe^2+^, Fe^3+^, Co^2+^, Co^3+^ and Ni^2+^; Lord *et al.*, 2009 ▸). All five-membered chelate rings of the tacn ligands have the stable gauche conformations. Three tetra­hedral [ZnCl_4_]^2−^ anions and an additional water mol­ecule remain outside the coordination sphere of Cr^3+^. Each ZnCl_4_^2−^ anion has a slightly distorted tetra­hedral coordination geometry because of the influence of hydrogen bonding on the Zn—Cl lengths and the Cl—Zn–Cl angles. The Zn—Cl bond lengths involved in hydrogen bonds were all found to have longer bonds than those not involved.

## Supra­molecular features   

**Figure 2:**
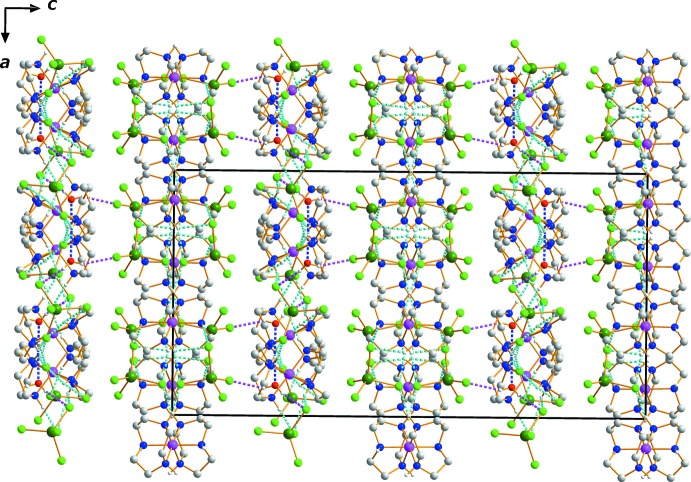
The crystal packing of complex (I) viewed perpendicular to the *ac* plane. Dashed lines represent O—H⋯Cl (purple), N—H⋯O (blue) and N—H⋯Cl (cyan) hydrogen-bonding inter­actions.

Extensive hydrogen-bonding inter­actions occur in the crystal structure (Table 1 ▸). The supra­molecular architecture involves hydrogen-bonding inter­actions with the N—H groups from each of the tacn ligands, the O—H groups of the lattice water mol­ecules acting as donors, and Cl atoms of the [ZnCl_4_]^2−^ anions and the O atoms of the water mol­ecules acting as acceptors, giving rise to a three-dimensional network structure. The network comprises columns of mol­ecules that form along the *a-*axis direction (Fig. 2 ▸). These hydrogen-bonded networks help to stabilize the crystal structure.

## Database survey   

A search of the Cambridge Structural Database (Version 5.39, Aug 2018 with four updates; Groom *et al.*, 2016 ▸) gave 11 hits for trivalent metal complexes containing two tacn (C_6_H_15_N_3_) ligands. The structures of [Ni(tacn)_2_](NO_3_)Cl·H_2_O (Zompa & Margulis, 1978 ▸), [Fe(tacn)_2_]Cl_3_·5H_2_O (Boeyens *et al.*, 1985 ▸), [Pd(tacn)_2_](PF_6_)_3_ (Blake *et al.*, 1988 ▸) and [Co(tacn)_2_](ClO_4_)_3_ (Wang *et al.*, 2002 ▸) have been published previously. However, only one structure containing the [Cr(tacn)_3_]^3+^ form is present (Scarborough *et al.*, 2011 ▸). Each metal ion in all of these complexes is sandwiched between two tridentate tacn macrocycles. Until now, no structure of any salt of [Cr(tacn)_2_]^3+^ with the [ZnCl_4_]^2−^ anion has been deposited.

## Synthesis and crystallization   

Commercially available (Sigma–Aldrich) 1,4,7-tri­aza­cyclo­nonane was used as provided. All other chemicals were the best AR grade available. The starting material [Cr(tacn)_2_]Cl_3_ was prepared according to the literature (Wieghardt *et al.*, 1983 ▸). The crude trichloride salt (0.10 g) was dissolved in 7 mL of 0.5 *M* HCl at 313 K. 5 mL of a 1 *M* HCl solution containing 0.25 g of solid ZnCl_2_ were added to this solution. The resulting mixture was filtered, and allowed to stand at room temperature for two days to give plate-like yellow crystals of the title tetra­chlorido­zincate(II) salt suitable for single-crystal X-ray diffraction.

## Refinement   

Crystal data, data collection and structure refinement details are summarized in Table 2 ▸. Non-hydrogen atoms were refined anisotropically. All H atoms were placed in geometrically idealized positions and constrained to ride on their parent atoms, with C—H = 0.99 Å and N—H = 1.00 Å, and with *U*_iso_(H) values of 1.2*U*_eq_ of the parent atoms. The O-bound H atoms of the water mol­ecules were assigned based on a difference-Fourier map, and were refined with distance restraints of 0.95 (10) Å (using the DFIX and DANG commands), and *U*_iso_(H) values of 1.5*U*_eq_ of the oxygen atom.

## Supplementary Material

Crystal structure: contains datablock(s) I. DOI: 10.1107/S2056989019003086/sj5569sup1.cif

Structure factors: contains datablock(s) I. DOI: 10.1107/S2056989019003086/sj5569Isup2.hkl

CCDC reference: 1900397

Additional supporting information:  crystallographic information; 3D view; checkCIF report

## Figures and Tables

**Table 1:** Hydrogen-bond geometry (Å, °)

*D*—H⋯*A*	*D*—H	H⋯*A*	*D*⋯*A*	*D*—H⋯*A*
N5*A*—H5*A*⋯O1*W*	1.00	2.46	3.1535 (19)	126
N2*B*—H2*B*⋯Cl1*E*	1.00	2.25	3.1608 (12)	151
N4*B*—H4*B*⋯Cl2*D*	1.00	2.24	3.1179 (12)	146
O1*W*—H2*OW*⋯Cl3*D*	0.96 (1)	2.44 (1)	3.3163 (16)	152 (2)
N1*A*—H1*A*⋯Cl4*C* ^i^	1.00	2.23	3.2091 (13)	167
N4*A*—H4*A*⋯Cl1*C* ^i^	1.00	2.29	3.2377 (12)	158
N2*A*—H2*A*⋯Cl2*C* ^ii^	1.00	2.42	3.2981 (13)	146
N6*A*—H6*A*⋯Cl4*C* ^ii^	1.00	2.23	3.1811 (13)	159
N3*A*—H3*A*⋯Cl1*C* ^iii^	1.00	2.62	3.4416 (13)	140
N5*A*—H5*A*⋯Cl1*C* ^iii^	1.00	2.50	3.2875 (13)	136
N1*B*—H1*B*⋯Cl2*D* ^iv^	1.00	2.42	3.2707 (12)	143
N3*B*—H3*B*⋯Cl4*E* ^iv^	1.00	2.36	3.2884 (12)	154
N5*B*—H5*B*⋯Cl1*E* ^iv^	1.00	2.46	3.2932 (12)	141
N6*B*—H6*B*⋯Cl4*D* ^iv^	1.00	2.35	3.2935 (12)	157
O1*W*—H1*OW*⋯Cl2*C* ^v^	0.95 (1)	2.32 (1)	3.2520 (15)	166 (2)

Symmetry codes: (i) 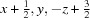; (ii) 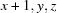; (iii) 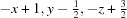; (iv) 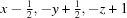; (v) 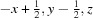.

**Table 2:** Experimental details

Crystal data	
Chemical formula	[Cr(C_6_H_15_N_3_)_2_]_2_[ZnCl_4_]_3_·H_2_O
*M*_r_	1260.36
Crystal system, space group	Orthorhombic, *P* *b* *c* *a*
Temperature (K)	100
*a*, *b*, *c* (Å)	17.281 (4), 16.753 (3), 33.405 (7)
*V* (Å^3^)	9671 (3)
*Z*	8
Radiation type	Synchrotron, λ = 0.62998 Å
μ (mm^−1^)	1.86
Crystal size (mm)	0.15 × 0.10 × 0.08

Data collection	
Diffractometer	ADSC Q210 CCD area detector
Absorption correction	Empirical (using intensity measurements) (*HKL3000sm *SCALEPACK**; Otwinowski et al., 1997 ▸)
*T*_min_, *T*_max_	0.768, 1.000
No. of measured, independent and observed [*I* > 2σ(*I*)] reflections	95478, 13601, 12445
*R*_int_	0.050
(sin θ/λ)_max_ (Å^−1^)	0.696

Refinement	
*R*[*F* ^2^ > 2σ(*F* ^2^)], *wR*(*F* ^2^), *S*	0.025, 0.071, 1.06
No. of reflections	13601
No. of parameters	493
No. of restraints	3
H-atom treatment	H atoms treated by a mixture of independent and constrained refinement
Δρ_max_, Δρ_min_ (e Å^−3^)	1.01, −0.96

Computer programs: *PAL BL2D-SMDC* (Shin *et al.*, 2016 ▸), *HKL3000sm* (Otwinowski & Minor, 1997 ▸), *SHELXT2018* (Sheldrick, 2015*a* ▸), *SHELXL2018* (Sheldrick, 2015*b* ▸), *DIAMOND* (Putz & Brandenburg, 2014 ▸) and *publCIF* (Westrip, 2010 ▸).

## supplementary crystallographic information

### Crystal data

**Table:**

[Cr(C_6_H_15_N_3_)_2_]_2_[ZnCl_4_]_3_·H_2_O	*D*_x_ = 1.731 Mg m^−^^3^
*M**_r_* = 1260.36	Synchrotron radiation, λ = 0.62998 Å
Orthorhombic, *P**b**c**a*	Cell parameters from 295495 reflections
*a* = 17.281 (4) Å	θ = 0.4–33.6°
*b* = 16.753 (3) Å	µ = 1.86 mm^−^^1^
*c* = 33.405 (7) Å	*T* = 100 K
*V* = 9671 (3) Å^3^	Plate, yellow
*Z* = 8	0.15 × 0.10 × 0.08 mm
*F*(000) = 5120	

### Data collection

**Table:**

ADSC Q210 CCD area detector diffractometer	12445 reflections with *I* > 2σ(*I*)
Radiation source: PLSII 2D bending magnet	*R*_int_ = 0.050
ω scan	θ_max_ = 26.0°, θ_min_ = 1.6°
Absorption correction: empirical (using intensity measurements) (*HKL3000sm SCALEPACK*; Otwinowski et al., 1997)	*h* = −24→24
*T*_min_ = 0.768, *T*_max_ = 1.000	*k* = −23→23
95478 measured reflections	*l* = −46→46
13601 independent reflections	

### Refinement

**Table:**

Refinement on *F*^2^	3 restraints
Least-squares matrix: full	Hydrogen site location: mixed
*R*[*F*^2^ > 2σ(*F*^2^)] = 0.025	H atoms treated by a mixture of independent and constrained refinement
*wR*(*F*^2^) = 0.071	*w* = 1/[σ^2^(*F*_o_^2^) + (0.0409*P*)^2^ + 5.110*P*] where *P* = (*F*_o_^2^ + 2*F*_c_^2^)/3
*S* = 1.06	(Δ/σ)_max_ = 0.006
13601 reflections	Δρ_max_ = 1.01 e Å^−^^3^
493 parameters	Δρ_min_ = −0.96 e Å^−^^3^

### Special details

**Table:**

Geometry. All esds (except the esd in the dihedral angle between two l.s. planes) are estimated using the full covariance matrix. The cell esds are taken into account individually in the estimation of esds in distances, angles and torsion angles; correlations between esds in cell parameters are only used when they are defined by crystal symmetry. An approximate (isotropic) treatment of cell esds is used for estimating esds involving l.s. planes.

### Fractional atomic coordinates and isotropic or equivalent isotropic displacement parameters (Å^2^)

**Table:**

	*x*	*y*	*z*	*U*_iso_*/*U*_eq_	
Cr1A	0.82954 (2)	0.48430 (2)	0.74710 (2)	0.00703 (4)	
N1A	0.73238 (6)	0.48954 (7)	0.78330 (3)	0.0113 (2)	
H1A	0.687279	0.506644	0.766621	0.014*	
N2A	0.87599 (6)	0.55581 (6)	0.79209 (3)	0.0112 (2)	
H2A	0.915224	0.592659	0.780136	0.013*	
N3A	0.86016 (7)	0.39319 (6)	0.78636 (3)	0.0116 (2)	
H3A	0.874864	0.344914	0.770486	0.014*	
N4A	0.79614 (7)	0.57390 (6)	0.70780 (3)	0.01109 (19)	
H4A	0.764457	0.614134	0.722638	0.013*	
N5A	0.78444 (7)	0.41078 (7)	0.70289 (3)	0.0131 (2)	
H5A	0.761936	0.362150	0.715765	0.016*	
N6A	0.92602 (6)	0.48125 (7)	0.70986 (3)	0.0126 (2)	
H6A	0.973683	0.486302	0.726647	0.015*	
C1A	0.74241 (8)	0.54831 (8)	0.81699 (4)	0.0149 (2)	
H1A1	0.752269	0.519256	0.842286	0.018*	
H1A2	0.694404	0.579882	0.820294	0.018*	
C2A	0.80979 (8)	0.60390 (8)	0.80808 (4)	0.0152 (2)	
H2A1	0.793951	0.644436	0.788136	0.018*	
H2A2	0.825747	0.631907	0.832836	0.018*	
C3A	0.91411 (8)	0.50600 (8)	0.82401 (4)	0.0153 (2)	
H3A1	0.880148	0.503350	0.847837	0.018*	
H3A2	0.963528	0.531163	0.832107	0.018*	
C4A	0.92954 (8)	0.42221 (9)	0.80863 (4)	0.0161 (3)	
H4A1	0.975126	0.422542	0.790678	0.019*	
H4A2	0.940669	0.386092	0.831365	0.019*	
C5A	0.79365 (8)	0.37205 (8)	0.81358 (4)	0.0141 (2)	
H5A1	0.803434	0.393743	0.840678	0.017*	
H5A2	0.789349	0.313284	0.815748	0.017*	
C6A	0.71858 (8)	0.40602 (8)	0.79736 (4)	0.0147 (2)	
H6A1	0.699861	0.372786	0.774846	0.018*	
H6A2	0.678589	0.405796	0.818579	0.018*	
C7A	0.74887 (8)	0.54068 (8)	0.67381 (4)	0.0159 (2)	
H7A1	0.780731	0.539014	0.649191	0.019*	
H7A2	0.703905	0.575871	0.668727	0.019*	
C8A	0.72081 (8)	0.45739 (9)	0.68380 (4)	0.0171 (3)	
H8A1	0.676127	0.460616	0.702275	0.021*	
H8A2	0.703626	0.430144	0.659046	0.021*	
C9A	0.84575 (9)	0.38539 (8)	0.67355 (4)	0.0170 (3)	
H9A1	0.839435	0.415422	0.648233	0.020*	
H9A2	0.839895	0.327834	0.667553	0.020*	
C10A	0.92552 (9)	0.40078 (8)	0.69058 (4)	0.0173 (3)	
H10C	0.938524	0.359294	0.710587	0.021*	
H10D	0.964601	0.398797	0.668937	0.021*	
C11A	0.92473 (8)	0.54815 (8)	0.67986 (4)	0.0150 (2)	
H11A	0.908068	0.527532	0.653463	0.018*	
H11B	0.977349	0.570786	0.676931	0.018*	
C12A	0.86932 (9)	0.61256 (8)	0.69379 (4)	0.0149 (2)	
H12C	0.892906	0.643434	0.715918	0.018*	
H12D	0.858029	0.649697	0.671493	0.018*	
Cr2B	0.37785 (2)	0.24725 (2)	0.50148 (2)	0.00596 (4)	
N1B	0.30089 (6)	0.15782 (6)	0.51771 (3)	0.00828 (18)	
H1B	0.262173	0.150115	0.495797	0.010*	
N2B	0.45967 (6)	0.16291 (7)	0.51723 (3)	0.01044 (19)	
H2B	0.505894	0.169844	0.499566	0.013*	
N3B	0.37847 (6)	0.26919 (7)	0.56267 (3)	0.00966 (19)	
H3B	0.365415	0.326551	0.567453	0.012*	
N4B	0.45940 (6)	0.33063 (7)	0.48468 (3)	0.01023 (19)	
H4B	0.506540	0.322634	0.501568	0.012*	
N5B	0.30051 (6)	0.33765 (6)	0.48693 (3)	0.00844 (18)	
H5B	0.262920	0.344899	0.509396	0.010*	
N6B	0.37491 (6)	0.22685 (7)	0.43992 (3)	0.00947 (19)	
H6B	0.360772	0.169847	0.434887	0.011*	
C1B	0.34251 (7)	0.08032 (7)	0.52523 (4)	0.0118 (2)	
H1B1	0.313751	0.036070	0.512406	0.014*	
H1B2	0.344478	0.069835	0.554383	0.014*	
C2B	0.42428 (8)	0.08337 (8)	0.50860 (4)	0.0136 (2)	
H2B1	0.455922	0.040725	0.520962	0.016*	
H2B2	0.423093	0.074245	0.479324	0.016*	
C3B	0.48497 (8)	0.17292 (8)	0.56018 (4)	0.0137 (2)	
H3B1	0.541986	0.168265	0.561918	0.016*	
H3B2	0.461894	0.130150	0.576784	0.016*	
C4B	0.45967 (7)	0.25416 (8)	0.57609 (4)	0.0130 (2)	
H4B1	0.462384	0.254683	0.605687	0.016*	
H4B2	0.494325	0.296402	0.565691	0.016*	
C5B	0.32037 (7)	0.21759 (8)	0.58400 (4)	0.0116 (2)	
H5B1	0.294559	0.248872	0.605307	0.014*	
H5B2	0.347052	0.171820	0.596692	0.014*	
C6B	0.26045 (7)	0.18728 (8)	0.55445 (4)	0.0103 (2)	
H6B1	0.230193	0.143422	0.566681	0.012*	
H6B2	0.224320	0.230879	0.547310	0.012*	
C7B	0.42574 (8)	0.41035 (8)	0.49455 (4)	0.0132 (2)	
H7B1	0.456972	0.452904	0.481828	0.016*	
H7B2	0.426943	0.418697	0.523886	0.016*	
C8B	0.34263 (7)	0.41501 (7)	0.47962 (4)	0.0118 (2)	
H8B1	0.315365	0.458898	0.493589	0.014*	
H8B2	0.342533	0.427027	0.450607	0.014*	
C9B	0.25820 (7)	0.30974 (8)	0.45055 (4)	0.0111 (2)	
H9B1	0.227682	0.354205	0.439070	0.013*	
H9B2	0.222138	0.266153	0.457783	0.013*	
C10B	0.31657 (7)	0.28003 (8)	0.42001 (4)	0.0118 (2)	
H10A	0.289480	0.250080	0.398651	0.014*	
H10B	0.343222	0.326058	0.407548	0.014*	
C11B	0.45521 (7)	0.24149 (8)	0.42513 (4)	0.0124 (2)	
H11C	0.455822	0.242191	0.395500	0.015*	
H11D	0.490099	0.198424	0.434418	0.015*	
C12B	0.48249 (8)	0.32173 (8)	0.44140 (4)	0.0130 (2)	
H12A	0.539462	0.325548	0.438979	0.016*	
H12B	0.459285	0.365434	0.425469	0.016*	
Zn1C	0.07329 (2)	0.67835 (2)	0.74676 (2)	0.01331 (4)	
Cl1C	0.19073 (2)	0.72564 (2)	0.76712 (2)	0.01642 (7)	
Cl2C	−0.02687 (2)	0.72432 (2)	0.78306 (2)	0.02366 (8)	
Cl3C	0.06377 (3)	0.71107 (2)	0.68204 (2)	0.02680 (9)	
Cl4C	0.07302 (2)	0.54414 (2)	0.75772 (2)	0.01610 (7)	
Zn2D	0.65175 (2)	0.39591 (2)	0.58225 (2)	0.01018 (4)	
Cl1D	0.57342 (2)	0.49024 (2)	0.60669 (2)	0.02659 (9)	
Cl2D	0.62528 (2)	0.36622 (2)	0.51676 (2)	0.01529 (6)	
Cl3D	0.64207 (2)	0.28477 (2)	0.62022 (2)	0.01465 (6)	
Cl4D	0.77816 (2)	0.43965 (2)	0.57568 (2)	0.01200 (6)	
Zn3E	0.65313 (2)	0.10971 (2)	0.41217 (2)	0.00947 (4)	
Cl1E	0.62418 (2)	0.13302 (2)	0.47869 (2)	0.01281 (6)	
Cl2E	0.64032 (2)	0.22326 (2)	0.37751 (2)	0.01410 (6)	
Cl3E	0.57773 (2)	0.01372 (2)	0.38615 (2)	0.02218 (8)	
Cl4E	0.78037 (2)	0.06714 (2)	0.41790 (2)	0.01168 (6)	
O1W	0.62401 (9)	0.32554 (8)	0.71692 (4)	0.0364 (3)	
H1OW	0.5882 (13)	0.2988 (15)	0.7337 (6)	0.055*	
H2OW	0.6101 (14)	0.3130 (15)	0.6899 (3)	0.055*	

### Atomic displacement parameters (Å^2^)

**Table:**

	*U*^11^	*U*^22^	*U*^33^	*U*^12^	*U*^13^	*U*^23^
Cr1A	0.00877 (10)	0.00634 (9)	0.00597 (9)	0.00020 (7)	0.00093 (6)	0.00002 (6)
N1A	0.0098 (5)	0.0151 (5)	0.0089 (5)	0.0009 (4)	0.0007 (4)	−0.0011 (4)
N2A	0.0135 (5)	0.0105 (5)	0.0097 (5)	−0.0015 (4)	−0.0022 (4)	0.0003 (4)
N3A	0.0149 (5)	0.0089 (5)	0.0110 (5)	0.0010 (4)	0.0036 (4)	0.0025 (4)
N4A	0.0155 (5)	0.0095 (4)	0.0083 (4)	0.0034 (4)	0.0001 (4)	−0.0006 (4)
N5A	0.0183 (5)	0.0110 (5)	0.0101 (5)	−0.0032 (4)	0.0027 (4)	−0.0027 (4)
N6A	0.0128 (5)	0.0115 (5)	0.0134 (5)	0.0017 (4)	0.0041 (4)	0.0023 (4)
C1A	0.0173 (6)	0.0166 (6)	0.0107 (5)	0.0043 (5)	0.0018 (5)	−0.0042 (5)
C2A	0.0230 (7)	0.0109 (5)	0.0117 (6)	0.0031 (5)	−0.0009 (5)	−0.0039 (4)
C3A	0.0154 (6)	0.0177 (6)	0.0128 (6)	−0.0013 (5)	−0.0054 (5)	0.0037 (5)
C4A	0.0134 (6)	0.0174 (6)	0.0174 (6)	0.0024 (5)	−0.0020 (5)	0.0075 (5)
C5A	0.0178 (6)	0.0129 (5)	0.0115 (5)	−0.0023 (5)	0.0044 (5)	0.0030 (4)
C6A	0.0141 (6)	0.0174 (6)	0.0126 (6)	−0.0050 (5)	0.0030 (5)	0.0006 (5)
C7A	0.0186 (6)	0.0189 (6)	0.0104 (5)	0.0028 (5)	−0.0037 (5)	−0.0008 (5)
C8A	0.0166 (6)	0.0227 (7)	0.0120 (6)	−0.0034 (5)	−0.0017 (5)	−0.0036 (5)
C9A	0.0284 (7)	0.0103 (5)	0.0124 (6)	0.0005 (5)	0.0074 (5)	−0.0030 (4)
C10A	0.0231 (7)	0.0117 (6)	0.0171 (6)	0.0074 (5)	0.0089 (5)	0.0015 (5)
C11A	0.0180 (6)	0.0119 (6)	0.0152 (6)	−0.0019 (5)	0.0062 (5)	0.0038 (5)
C12A	0.0237 (7)	0.0076 (5)	0.0134 (6)	−0.0016 (5)	0.0017 (5)	0.0023 (4)
Cr2B	0.00346 (9)	0.00755 (9)	0.00689 (9)	0.00014 (6)	0.00000 (6)	0.00075 (6)
N1B	0.0063 (4)	0.0082 (4)	0.0103 (4)	0.0000 (4)	0.0001 (4)	0.0014 (3)
N2B	0.0066 (5)	0.0135 (5)	0.0112 (5)	0.0028 (4)	0.0004 (4)	0.0017 (4)
N3B	0.0070 (5)	0.0129 (5)	0.0090 (5)	−0.0008 (4)	−0.0002 (4)	−0.0006 (4)
N4B	0.0062 (4)	0.0137 (5)	0.0107 (5)	−0.0031 (4)	−0.0007 (4)	0.0018 (4)
N5B	0.0063 (4)	0.0089 (4)	0.0101 (4)	0.0007 (4)	0.0005 (4)	0.0011 (4)
N6B	0.0075 (5)	0.0118 (5)	0.0091 (4)	0.0003 (4)	0.0003 (4)	−0.0012 (4)
C1B	0.0117 (6)	0.0086 (5)	0.0151 (6)	0.0019 (4)	−0.0007 (4)	0.0020 (4)
C2B	0.0128 (6)	0.0106 (5)	0.0173 (6)	0.0048 (5)	0.0017 (5)	0.0002 (5)
C3B	0.0085 (5)	0.0204 (6)	0.0121 (5)	0.0021 (5)	−0.0017 (4)	0.0036 (5)
C4B	0.0078 (5)	0.0199 (6)	0.0112 (5)	−0.0031 (5)	−0.0022 (4)	0.0015 (5)
C5B	0.0085 (5)	0.0169 (6)	0.0093 (5)	−0.0017 (4)	0.0019 (4)	0.0014 (4)
C6B	0.0055 (5)	0.0139 (5)	0.0116 (5)	−0.0002 (4)	0.0019 (4)	0.0008 (4)
C7B	0.0137 (6)	0.0107 (5)	0.0153 (6)	−0.0050 (5)	−0.0002 (5)	−0.0006 (4)
C8B	0.0123 (6)	0.0082 (5)	0.0151 (6)	−0.0011 (4)	0.0010 (4)	0.0021 (4)
C9B	0.0058 (5)	0.0152 (5)	0.0121 (5)	0.0004 (4)	−0.0019 (4)	0.0008 (4)
C10B	0.0086 (5)	0.0168 (6)	0.0100 (5)	0.0015 (4)	−0.0016 (4)	0.0009 (4)
C11B	0.0077 (5)	0.0176 (6)	0.0119 (5)	0.0019 (5)	0.0029 (4)	0.0001 (4)
C12B	0.0089 (5)	0.0185 (6)	0.0116 (5)	−0.0023 (4)	0.0019 (4)	0.0021 (4)
Zn1C	0.01218 (8)	0.00967 (8)	0.01807 (8)	0.00023 (6)	0.00177 (5)	0.00160 (5)
Cl1C	0.01393 (14)	0.00883 (13)	0.02650 (17)	0.00090 (10)	−0.00337 (12)	0.00088 (11)
Cl2C	0.01734 (16)	0.01496 (15)	0.0387 (2)	−0.00191 (12)	0.00984 (14)	−0.00832 (14)
Cl3C	0.0391 (2)	0.02137 (17)	0.01994 (17)	−0.01329 (15)	−0.00752 (15)	0.00866 (13)
Cl4C	0.01283 (15)	0.01041 (14)	0.02507 (16)	0.00074 (11)	0.00208 (12)	0.00425 (11)
Zn2D	0.00835 (7)	0.01209 (7)	0.01009 (7)	0.00124 (5)	0.00181 (5)	0.00072 (5)
Cl1D	0.0306 (2)	0.02487 (18)	0.02429 (18)	0.01727 (15)	0.01784 (15)	0.01057 (14)
Cl2D	0.00648 (13)	0.02672 (17)	0.01266 (14)	−0.00212 (11)	−0.00121 (10)	−0.00219 (11)
Cl3D	0.01435 (14)	0.01330 (14)	0.01629 (14)	−0.00026 (11)	0.00357 (11)	0.00350 (11)
Cl4D	0.01005 (13)	0.01445 (13)	0.01150 (13)	−0.00155 (10)	0.00138 (10)	−0.00169 (10)
Zn3E	0.00872 (7)	0.01067 (7)	0.00903 (7)	−0.00129 (5)	−0.00122 (5)	0.00038 (5)
Cl1E	0.00677 (13)	0.02058 (15)	0.01107 (13)	0.00090 (11)	0.00149 (10)	−0.00140 (11)
Cl2E	0.01364 (13)	0.01323 (13)	0.01543 (14)	0.00019 (11)	−0.00224 (11)	0.00407 (10)
Cl3E	0.02649 (18)	0.01875 (16)	0.02131 (16)	−0.01179 (13)	−0.01415 (14)	0.00553 (12)
Cl4E	0.00968 (13)	0.01421 (13)	0.01113 (12)	0.00120 (10)	0.00010 (10)	−0.00091 (10)
O1W	0.0434 (8)	0.0350 (7)	0.0307 (7)	−0.0181 (6)	−0.0078 (6)	−0.0003 (6)

### Geometric parameters (Å, º)

**Table:**

Cr1A—N1A	2.0709 (11)	N1B—C1B	1.5053 (16)
Cr1A—N5A	2.0750 (11)	N1B—H1B	1.0000
Cr1A—N4A	2.0761 (11)	N2B—C2B	1.4942 (17)
Cr1A—N6A	2.0807 (11)	N2B—C3B	1.5092 (17)
Cr1A—N3A	2.0808 (11)	N2B—H2B	1.0000
Cr1A—N2A	2.0828 (11)	N3B—C4B	1.4943 (16)
N1A—C6A	1.4951 (17)	N3B—C5B	1.5043 (16)
N1A—C1A	1.5053 (17)	N3B—H3B	1.0000
N1A—H1A	1.0000	N4B—C7B	1.4936 (17)
N2A—C2A	1.4977 (17)	N4B—C12B	1.5070 (17)
N2A—C3A	1.5058 (17)	N4B—H4B	1.0000
N2A—H2A	1.0000	N5B—C9B	1.4933 (16)
N3A—C4A	1.4923 (18)	N5B—C8B	1.5064 (16)
N3A—C5A	1.5076 (17)	N5B—H5B	1.0000
N3A—H3A	1.0000	N6B—C11B	1.4933 (16)
N4A—C12A	1.4958 (18)	N6B—C10B	1.5008 (16)
N4A—C7A	1.5053 (17)	N6B—H6B	1.0000
N4A—H4A	1.0000	C1B—C2B	1.5193 (18)
N5A—C8A	1.4919 (18)	C1B—H1B1	0.9900
N5A—C9A	1.5048 (17)	C1B—H1B2	0.9900
N5A—H5A	1.0000	C2B—H2B1	0.9900
N6A—C10A	1.4942 (18)	C2B—H2B2	0.9900
N6A—C11A	1.5036 (17)	C3B—C4B	1.525 (2)
N6A—H6A	1.0000	C3B—H3B1	0.9900
C1A—C2A	1.520 (2)	C3B—H3B2	0.9900
C1A—H1A1	0.9900	C4B—H4B1	0.9900
C1A—H1A2	0.9900	C4B—H4B2	0.9900
C2A—H2A1	0.9900	C5B—C6B	1.5181 (18)
C2A—H2A2	0.9900	C5B—H5B1	0.9900
C3A—C4A	1.518 (2)	C5B—H5B2	0.9900
C3A—H3A1	0.9900	C6B—H6B1	0.9900
C3A—H3A2	0.9900	C6B—H6B2	0.9900
C4A—H4A1	0.9900	C7B—C8B	1.5224 (19)
C4A—H4A2	0.9900	C7B—H7B1	0.9900
C5A—C6A	1.5168 (19)	C7B—H7B2	0.9900
C5A—H5A1	0.9900	C8B—H8B1	0.9900
C5A—H5A2	0.9900	C8B—H8B2	0.9900
C6A—H6A1	0.9900	C9B—C10B	1.5184 (18)
C6A—H6A2	0.9900	C9B—H9B1	0.9900
C7A—C8A	1.514 (2)	C9B—H9B2	0.9900
C7A—H7A1	0.9900	C10B—H10A	0.9900
C7A—H7A2	0.9900	C10B—H10B	0.9900
C8A—H8A1	0.9900	C11B—C12B	1.5247 (19)
C8A—H8A2	0.9900	C11B—H11C	0.9900
C9A—C10A	1.513 (2)	C11B—H11D	0.9900
C9A—H9A1	0.9900	C12B—H12A	0.9900
C9A—H9A2	0.9900	C12B—H12B	0.9900
C10A—H10C	0.9900	Zn1C—Cl3C	2.2365 (6)
C10A—H10D	0.9900	Zn1C—Cl2C	2.2492 (5)
C11A—C12A	1.5159 (19)	Zn1C—Cl4C	2.2780 (6)
C11A—H11A	0.9900	Zn1C—Cl1C	2.2825 (5)
C11A—H11B	0.9900	Zn2D—Cl1D	2.2353 (5)
C12A—H12C	0.9900	Zn2D—Cl3D	2.2592 (5)
C12A—H12D	0.9900	Zn2D—Cl2D	2.2896 (6)
Cr2B—N4B	2.0621 (11)	Zn2D—Cl4D	2.3145 (5)
Cr2B—N2B	2.0670 (11)	Zn3E—Cl2E	2.2379 (5)
Cr2B—N1B	2.0755 (11)	Zn3E—Cl3E	2.2447 (5)
Cr2B—N3B	2.0772 (12)	Zn3E—Cl1E	2.3112 (6)
Cr2B—N5B	2.0776 (11)	Zn3E—Cl4E	2.3195 (5)
Cr2B—N6B	2.0851 (11)	O1W—H1OW	0.947 (9)
N1B—C6B	1.4962 (16)	O1W—H2OW	0.956 (9)
			
N1A—Cr1A—N5A	97.83 (5)	N4B—Cr2B—N6B	81.91 (4)
N1A—Cr1A—N4A	96.50 (5)	N2B—Cr2B—N6B	98.96 (4)
N5A—Cr1A—N4A	82.79 (5)	N1B—Cr2B—N6B	97.11 (4)
N1A—Cr1A—N6A	178.59 (5)	N3B—Cr2B—N6B	178.66 (4)
N5A—Cr1A—N6A	82.00 (5)	N5B—Cr2B—N6B	82.71 (4)
N4A—Cr1A—N6A	82.09 (5)	C6B—N1B—C1B	111.76 (10)
N1A—Cr1A—N3A	82.49 (4)	C6B—N1B—Cr2B	105.99 (7)
N5A—Cr1A—N3A	96.24 (5)	C1B—N1B—Cr2B	111.10 (8)
N4A—Cr1A—N3A	178.51 (5)	C6B—N1B—H1B	109.3
N6A—Cr1A—N3A	98.92 (5)	C1B—N1B—H1B	109.3
N1A—Cr1A—N2A	82.35 (5)	Cr2B—N1B—H1B	109.3
N5A—Cr1A—N2A	178.68 (5)	C2B—N2B—C3B	113.66 (10)
N4A—Cr1A—N2A	98.49 (5)	C2B—N2B—Cr2B	106.28 (8)
N6A—Cr1A—N2A	97.85 (5)	C3B—N2B—Cr2B	111.36 (8)
N3A—Cr1A—N2A	82.48 (5)	C2B—N2B—H2B	108.5
C6A—N1A—C1A	113.31 (10)	C3B—N2B—H2B	108.5
C6A—N1A—Cr1A	105.85 (8)	Cr2B—N2B—H2B	108.5
C1A—N1A—Cr1A	111.77 (8)	C4B—N3B—C5B	112.82 (10)
C6A—N1A—H1A	108.6	C4B—N3B—Cr2B	105.67 (8)
C1A—N1A—H1A	108.6	C5B—N3B—Cr2B	111.16 (8)
Cr1A—N1A—H1A	108.6	C4B—N3B—H3B	109.0
C2A—N2A—C3A	112.31 (10)	C5B—N3B—H3B	109.0
C2A—N2A—Cr1A	105.80 (8)	Cr2B—N3B—H3B	109.0
C3A—N2A—Cr1A	111.14 (8)	C7B—N4B—C12B	113.80 (10)
C2A—N2A—H2A	109.2	C7B—N4B—Cr2B	106.23 (8)
C3A—N2A—H2A	109.2	C12B—N4B—Cr2B	112.02 (8)
Cr1A—N2A—H2A	109.2	C7B—N4B—H4B	108.2
C4A—N3A—C5A	112.86 (11)	C12B—N4B—H4B	108.2
C4A—N3A—Cr1A	106.23 (8)	Cr2B—N4B—H4B	108.2
C5A—N3A—Cr1A	111.01 (8)	C9B—N5B—C8B	111.97 (10)
C4A—N3A—H3A	108.9	C9B—N5B—Cr2B	106.09 (8)
C5A—N3A—H3A	108.9	C8B—N5B—Cr2B	110.75 (8)
Cr1A—N3A—H3A	108.9	C9B—N5B—H5B	109.3
C12A—N4A—C7A	112.51 (10)	C8B—N5B—H5B	109.3
C12A—N4A—Cr1A	106.01 (8)	Cr2B—N5B—H5B	109.3
C7A—N4A—Cr1A	111.14 (8)	C11B—N6B—C10B	112.35 (10)
C12A—N4A—H4A	109.0	C11B—N6B—Cr2B	106.06 (8)
C7A—N4A—H4A	109.0	C10B—N6B—Cr2B	110.86 (8)
Cr1A—N4A—H4A	109.0	C11B—N6B—H6B	109.2
C8A—N5A—C9A	112.86 (11)	C10B—N6B—H6B	109.2
C8A—N5A—Cr1A	105.70 (8)	Cr2B—N6B—H6B	109.2
C9A—N5A—Cr1A	111.53 (9)	N1B—C1B—C2B	110.75 (10)
C8A—N5A—H5A	108.9	N1B—C1B—H1B1	109.5
C9A—N5A—H5A	108.9	C2B—C1B—H1B1	109.5
Cr1A—N5A—H5A	108.9	N1B—C1B—H1B2	109.5
C10A—N6A—C11A	112.65 (11)	C2B—C1B—H1B2	109.5
C10A—N6A—Cr1A	105.97 (8)	H1B1—C1B—H1B2	108.1
C11A—N6A—Cr1A	111.62 (8)	N2B—C2B—C1B	109.88 (10)
C10A—N6A—H6A	108.8	N2B—C2B—H2B1	109.7
C11A—N6A—H6A	108.8	C1B—C2B—H2B1	109.7
Cr1A—N6A—H6A	108.8	N2B—C2B—H2B2	109.7
N1A—C1A—C2A	110.02 (10)	C1B—C2B—H2B2	109.7
N1A—C1A—H1A1	109.7	H2B1—C2B—H2B2	108.2
C2A—C1A—H1A1	109.7	N2B—C3B—C4B	110.32 (10)
N1A—C1A—H1A2	109.7	N2B—C3B—H3B1	109.6
C2A—C1A—H1A2	109.7	C4B—C3B—H3B1	109.6
H1A1—C1A—H1A2	108.2	N2B—C3B—H3B2	109.6
N2A—C2A—C1A	108.99 (10)	C4B—C3B—H3B2	109.6
N2A—C2A—H2A1	109.9	H3B1—C3B—H3B2	108.1
C1A—C2A—H2A1	109.9	N3B—C4B—C3B	108.37 (10)
N2A—C2A—H2A2	109.9	N3B—C4B—H4B1	110.0
C1A—C2A—H2A2	109.9	C3B—C4B—H4B1	110.0
H2A1—C2A—H2A2	108.3	N3B—C4B—H4B2	110.0
N2A—C3A—C4A	110.46 (11)	C3B—C4B—H4B2	110.0
N2A—C3A—H3A1	109.6	H4B1—C4B—H4B2	108.4
C4A—C3A—H3A1	109.6	N3B—C5B—C6B	109.85 (10)
N2A—C3A—H3A2	109.6	N3B—C5B—H5B1	109.7
C4A—C3A—H3A2	109.6	C6B—C5B—H5B1	109.7
H3A1—C3A—H3A2	108.1	N3B—C5B—H5B2	109.7
N3A—C4A—C3A	109.19 (11)	C6B—C5B—H5B2	109.7
N3A—C4A—H4A1	109.8	H5B1—C5B—H5B2	108.2
C3A—C4A—H4A1	109.8	N1B—C6B—C5B	108.97 (10)
N3A—C4A—H4A2	109.8	N1B—C6B—H6B1	109.9
C3A—C4A—H4A2	109.8	C5B—C6B—H6B1	109.9
H4A1—C4A—H4A2	108.3	N1B—C6B—H6B2	109.9
N3A—C5A—C6A	110.40 (10)	C5B—C6B—H6B2	109.9
N3A—C5A—H5A1	109.6	H6B1—C6B—H6B2	108.3
C6A—C5A—H5A1	109.6	N4B—C7B—C8B	109.93 (10)
N3A—C5A—H5A2	109.6	N4B—C7B—H7B1	109.7
C6A—C5A—H5A2	109.6	C8B—C7B—H7B1	109.7
H5A1—C5A—H5A2	108.1	N4B—C7B—H7B2	109.7
N1A—C6A—C5A	109.08 (11)	C8B—C7B—H7B2	109.7
N1A—C6A—H6A1	109.9	H7B1—C7B—H7B2	108.2
C5A—C6A—H6A1	109.9	N5B—C8B—C7B	111.02 (10)
N1A—C6A—H6A2	109.9	N5B—C8B—H8B1	109.4
C5A—C6A—H6A2	109.9	C7B—C8B—H8B1	109.4
H6A1—C6A—H6A2	108.3	N5B—C8B—H8B2	109.4
N4A—C7A—C8A	110.37 (11)	C7B—C8B—H8B2	109.4
N4A—C7A—H7A1	109.6	H8B1—C8B—H8B2	108.0
C8A—C7A—H7A1	109.6	N5B—C9B—C10B	108.91 (10)
N4A—C7A—H7A2	109.6	N5B—C9B—H9B1	109.9
C8A—C7A—H7A2	109.6	C10B—C9B—H9B1	109.9
H7A1—C7A—H7A2	108.1	N5B—C9B—H9B2	109.9
N5A—C8A—C7A	109.90 (11)	C10B—C9B—H9B2	109.9
N5A—C8A—H8A1	109.7	H9B1—C9B—H9B2	108.3
C7A—C8A—H8A1	109.7	N6B—C10B—C9B	110.07 (10)
N5A—C8A—H8A2	109.7	N6B—C10B—H10A	109.6
C7A—C8A—H8A2	109.7	C9B—C10B—H10A	109.6
H8A1—C8A—H8A2	108.2	N6B—C10B—H10B	109.6
N5A—C9A—C10A	110.38 (11)	C9B—C10B—H10B	109.6
N5A—C9A—H9A1	109.6	H10A—C10B—H10B	108.2
C10A—C9A—H9A1	109.6	N6B—C11B—C12B	108.31 (10)
N5A—C9A—H9A2	109.6	N6B—C11B—H11C	110.0
C10A—C9A—H9A2	109.6	C12B—C11B—H11C	110.0
H9A1—C9A—H9A2	108.1	N6B—C11B—H11D	110.0
N6A—C10A—C9A	108.73 (11)	C12B—C11B—H11D	110.0
N6A—C10A—H10C	109.9	H11C—C11B—H11D	108.4
C9A—C10A—H10C	109.9	N4B—C12B—C11B	110.32 (10)
N6A—C10A—H10D	109.9	N4B—C12B—H12A	109.6
C9A—C10A—H10D	109.9	C11B—C12B—H12A	109.6
H10C—C10A—H10D	108.3	N4B—C12B—H12B	109.6
N6A—C11A—C12A	109.59 (10)	C11B—C12B—H12B	109.6
N6A—C11A—H11A	109.8	H12A—C12B—H12B	108.1
C12A—C11A—H11A	109.8	Cl3C—Zn1C—Cl2C	112.37 (2)
N6A—C11A—H11B	109.8	Cl3C—Zn1C—Cl4C	113.395 (16)
C12A—C11A—H11B	109.8	Cl2C—Zn1C—Cl4C	104.464 (16)
H11A—C11A—H11B	108.2	Cl3C—Zn1C—Cl1C	105.567 (16)
N4A—C12A—C11A	108.78 (10)	Cl2C—Zn1C—Cl1C	113.879 (19)
N4A—C12A—H12C	109.9	Cl4C—Zn1C—Cl1C	107.248 (14)
C11A—C12A—H12C	109.9	Cl1D—Zn2D—Cl3D	109.431 (19)
N4A—C12A—H12D	109.9	Cl1D—Zn2D—Cl2D	112.432 (17)
C11A—C12A—H12D	109.9	Cl3D—Zn2D—Cl2D	110.039 (18)
H12C—C12A—H12D	108.3	Cl1D—Zn2D—Cl4D	112.48 (2)
N4B—Cr2B—N2B	93.72 (5)	Cl3D—Zn2D—Cl4D	112.585 (14)
N4B—Cr2B—N1B	176.42 (4)	Cl2D—Zn2D—Cl4D	99.600 (13)
N2B—Cr2B—N1B	83.01 (5)	Cl2E—Zn3E—Cl3E	110.568 (18)
N4B—Cr2B—N3B	98.30 (4)	Cl2E—Zn3E—Cl1E	109.412 (16)
N2B—Cr2B—N3B	82.34 (4)	Cl3E—Zn3E—Cl1E	111.573 (16)
N1B—Cr2B—N3B	82.76 (4)	Cl2E—Zn3E—Cl4E	113.441 (14)
N4B—Cr2B—N5B	83.23 (5)	Cl3E—Zn3E—Cl4E	111.220 (19)
N2B—Cr2B—N5B	176.31 (4)	Cl1E—Zn3E—Cl4E	100.238 (13)
N1B—Cr2B—N5B	100.08 (5)	H1OW—O1W—H2OW	106.9 (17)
N3B—Cr2B—N5B	96.00 (4)		
			
C6A—N1A—C1A—C2A	136.07 (11)	C6B—N1B—C1B—C2B	−132.97 (11)
Cr1A—N1A—C1A—C2A	16.58 (13)	Cr2B—N1B—C1B—C2B	−14.81 (13)
C3A—N2A—C2A—C1A	−72.26 (13)	C3B—N2B—C2B—C1B	75.82 (13)
Cr1A—N2A—C2A—C1A	49.17 (11)	Cr2B—N2B—C2B—C1B	−47.02 (12)
N1A—C1A—C2A—N2A	−43.73 (14)	N1B—C1B—C2B—N2B	41.19 (14)
C2A—N2A—C3A—C4A	135.60 (12)	C2B—N2B—C3B—C4B	−134.90 (11)
Cr1A—N2A—C3A—C4A	17.29 (13)	Cr2B—N2B—C3B—C4B	−14.90 (12)
C5A—N3A—C4A—C3A	−73.41 (13)	C5B—N3B—C4B—C3B	71.42 (13)
Cr1A—N3A—C4A—C3A	48.46 (12)	Cr2B—N3B—C4B—C3B	−50.24 (11)
N2A—C3A—C4A—N3A	−43.79 (14)	N2B—C3B—C4B—N3B	43.29 (14)
C4A—N3A—C5A—C6A	134.71 (11)	C4B—N3B—C5B—C6B	−138.31 (11)
Cr1A—N3A—C5A—C6A	15.58 (13)	Cr2B—N3B—C5B—C6B	−19.81 (12)
C1A—N1A—C6A—C5A	−73.30 (13)	C1B—N1B—C6B—C5B	72.33 (13)
Cr1A—N1A—C6A—C5A	49.53 (11)	Cr2B—N1B—C6B—C5B	−48.85 (11)
N3A—C5A—C6A—N1A	−43.29 (14)	N3B—C5B—C6B—N1B	45.71 (13)
C12A—N4A—C7A—C8A	134.34 (12)	C12B—N4B—C7B—C8B	76.77 (13)
Cr1A—N4A—C7A—C8A	15.63 (13)	Cr2B—N4B—C7B—C8B	−46.95 (11)
C9A—N5A—C8A—C7A	−73.87 (13)	C9B—N5B—C8B—C7B	−131.91 (11)
Cr1A—N5A—C8A—C7A	48.28 (12)	Cr2B—N5B—C8B—C7B	−13.72 (12)
N4A—C7A—C8A—N5A	−42.73 (15)	N4B—C7B—C8B—N5B	40.46 (14)
C8A—N5A—C9A—C10A	134.71 (12)	C8B—N5B—C9B—C10B	72.14 (13)
Cr1A—N5A—C9A—C10A	15.90 (13)	Cr2B—N5B—C9B—C10B	−48.78 (11)
C11A—N6A—C10A—C9A	−72.64 (13)	C11B—N6B—C10B—C9B	−138.96 (11)
Cr1A—N6A—C10A—C9A	49.68 (12)	Cr2B—N6B—C10B—C9B	−20.48 (12)
N5A—C9A—C10A—N6A	−43.54 (14)	N5B—C9B—C10B—N6B	46.21 (13)
C10A—N6A—C11A—C12A	137.57 (12)	C10B—N6B—C11B—C12B	71.60 (13)
Cr1A—N6A—C11A—C12A	18.49 (13)	Cr2B—N6B—C11B—C12B	−49.67 (11)
C7A—N4A—C12A—C11A	−71.67 (13)	C7B—N4B—C12B—C11B	−134.99 (11)
Cr1A—N4A—C12A—C11A	50.00 (11)	Cr2B—N4B—C12B—C11B	−14.47 (13)
N6A—C11A—C12A—N4A	−45.37 (14)	N6B—C11B—C12B—N4B	42.52 (14)

### Hydrogen-bond geometry (Å, º)

**Table:**

*D*—H···*A*	*D*—H	H···*A*	*D*···*A*	*D*—H···*A*
N5*A*—H5*A*···O1*W*	1.00	2.46	3.1535 (19)	126
N2*B*—H2*B*···Cl1*E*	1.00	2.25	3.1608 (12)	151
N4*B*—H4*B*···Cl2*D*	1.00	2.24	3.1179 (12)	146
O1*W*—H2*OW*···Cl3*D*	0.96 (1)	2.44 (1)	3.3163 (16)	152 (2)
N1*A*—H1*A*···Cl4*C*^i^	1.00	2.23	3.2091 (13)	167
N4*A*—H4*A*···Cl1*C*^i^	1.00	2.29	3.2377 (12)	158
N2*A*—H2*A*···Cl2*C*^ii^	1.00	2.42	3.2981 (13)	146
N6*A*—H6*A*···Cl4*C*^ii^	1.00	2.23	3.1811 (13)	159
N3*A*—H3*A*···Cl1*C*^iii^	1.00	2.62	3.4416 (13)	140
N5*A*—H5*A*···Cl1*C*^iii^	1.00	2.50	3.2875 (13)	136
N1*B*—H1*B*···Cl2*D*^iv^	1.00	2.42	3.2707 (12)	143
N3*B*—H3*B*···Cl4*E*^iv^	1.00	2.36	3.2884 (12)	154
N5*B*—H5*B*···Cl1*E*^iv^	1.00	2.46	3.2932 (12)	141
N6*B*—H6*B*···Cl4*D*^iv^	1.00	2.35	3.2935 (12)	157
O1*W*—H1*OW*···Cl2*C*^v^	0.95 (1)	2.32 (1)	3.2520 (15)	166 (2)

Symmetry codes: (i) *x*+1/2, *y*, −*z*+3/2; (ii) *x*+1, *y*, *z*; (iii) −*x*+1, *y*−1/2, −*z*+3/2; (iv) *x*−1/2, −*y*+1/2, −*z*+1; (v) −*x*+1/2, *y*−1/2, *z*.
